# Methylene Blue Blocks and Reverses the Inhibitory Effect of Tau on PMCA Function

**DOI:** 10.3390/ijms20143521

**Published:** 2019-07-18

**Authors:** Maria Berrocal, Montaña Caballero-Bermejo, Carlos Gutierrez-Merino, Ana M. Mata

**Affiliations:** Departamento de Bioquímica y Biología Molecular y Genética, Facultad de Ciencias, Universidad de Extremadura and Instituto de Biomarcadores de Patologías Moleculares, Universidad de Extremadura, 06006 Badajoz, Spain

**Keywords:** methylene blue, tau, plasma membrane Ca^2+^-ATPase (PMCA), functional modulation, neurodegeneration

## Abstract

Methylene blue (MB) is a synthetic phenothiazine dye that, in the last years, has generated much debate about whether it could be a useful therapeutic drug for tau-related pathologies, such as Alzheimer’s disease (AD). However, the molecular mechanism of action is far from clear. Recently we reported that MB activates the plasma membrane Ca^2+^-ATPase (PMCA) in membranes from human and pig tissues and from cells cultures, and that it could protect against inactivation of PMCA by amyloid β-peptide (Aβ). The purpose of the present study is to further examine whether the MB could also modulate the inhibitory effect of tau, another key molecular marker of AD, on PMCA activity. By using kinetic assays in membranes from several tissues and cell cultures, we found that this phenothiazine was able to block and even to completely reverse the inhibitory effect of tau on PMCA. The results of this work point out that MB could mediate the toxic effect of tau related to the deregulation of calcium homeostasis by blocking the impairment of PMCA activity by tau. We then could conclude that MB could interfere with the toxic effects of tau by restoring the function of PMCA pump as a fine tuner of calcium homeostasis.

## 1. Introduction

Methylene blue (MB) is a thiophenazine dye, which has been extensively used in a wide range of applications, including as a bacteriological stain, as a redox analytical indicator, or as an antiseptic to treat several infections. In fact, MB is an FDA-approved drug used to treat a variety of diseases including malaria [1], fear and claustrophobia [2], and psychiatric disorders [3,4]. There is a growing biomedical interest in MB as a treatment strategy for diseases affecting the central nervous system because it can easily cross the blood-brain barrier [5,6]. In fact, it has been shown that MB produces a protective effect in ischemic brain damage [7,8], self-sustaining status epilepticus [9], Leber optic neuropathy [10], Alzheimer’s disease (AD) [11], Parkinson disease [12,13], and other neurodegenerative disorders [14]. Although the benefits of MB therapy in AD have been questioned, a recent study [11] has reported that a subgroup of patients who were treated with leuco-methylthioninium bis (hydromethanesulphonate) (a reduced form of MB) as a monotherapy showed a significant decrease in the rate of disease progression, compared to the placebo.

AD is a neurodegenerative disease characterized by a loss of neuronal synapses and progressive cognitive decline that is reaching epidemic proportions in an increasingly aging world population [15]. This explains the huge interest that exists to develop effective drugs to treat it. The presence of extracellular plaque deposits of amyloid β-peptide (Aβ) and intracellular neurofibrillary tangles (NFT) of tau protein are neuropathological hallmarks of neurodegeneration in AD [16,17,18]. On these grounds, the studies on the molecular mechanisms underlying the beneficial effect of MB in AD have mainly been focused on its role in preventing aggregation of several neurodegenerative proteins, including tau [19,20], and repairing mitochondrial dysfunction [21,22,23,24]. However, increasing experimental evidence suggests a link between tau and dysregulation of intracellular Ca^2+^ levels, via its interaction with receptors involved in the release of Ca^2+^ from intracellular stores or in the uptake of extracellular Ca^2+^ [25,26,27,28], and through its association with Ca^2+^-binding proteins [29]. P-type Ca^2+^-ATPase pumps are a family of plasma and intracellular membrane proteins actively involved in the fine-tuning of intracellular Ca^2+^ levels [30]. We have shown that the plasma membrane Ca^2+^-ATPase (PMCA) seems to be involved in Ca^2+^ dysregulation associated with AD, because it is functionally inhibited by both Aβ and tau [31,32]. In an effort to find compounds that can protect against this inhibition and/or even reverse it, we recently reported that calmodulin, a Ca^2+^-binding protein, was able to reverse and protect against the inhibitory effect of Aβ [33] and tau [34]. A similar effect was produced by MB against PMCA inhibition by Aβ, an effect mediated by the binding of MB to a site located in the C-terminal domain close to the last transmembrane helix of PMCA and different from calmodulin and Aβ binding sites in PMCA [35]. As noted in Berrocal et al. [35], this effect of MB is produced at micromolar concentrations, e.g., within the concentration range of MB reached in the nervous system in therapeutic bolus doses of MB, as shown in previous pharmacokinetic studies with rodents and humans [5,36,37].

In this work we show functional evidence of the protective effect of MB against toxicity induced by tau on PMCA function in human brain membranes prepared from control and AD-affected subjects, in pig brain purified preparation, and in cell cultures overexpressing specific PMCA isoforms. A protective role of MB against the toxic effect of exogenous tau addition on neuronal cultures is also shown. The effect of tau on the binding of MB to PMCA has been experimentally assessed by intrinsic fluorescence measurements.

## 2. Results

### 2.1. Methylene Blue (MB) Completely Reverses the Inhibitory Effects of Tau on Plasma Membrane Ca^2+^-ATPase (PMCA) Activity in Human Brain Membranes of Alzheimer’s Disease Patients and Control Subjects

**Figure 1:**
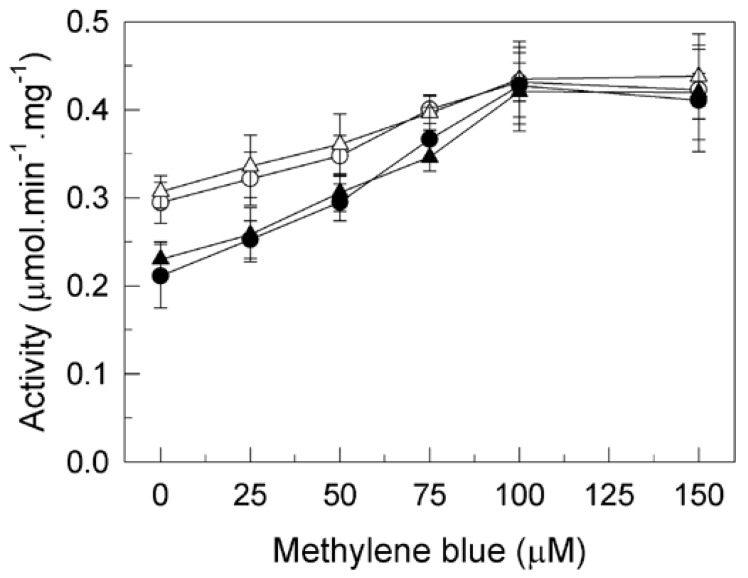
Effects of tau and/or methylene blue (MB) on Ca^2+^-ATPase activity in membranes from human control (HC, ⭘⬤) and Alzheimer’s disease (AD) affected (HAD, △▲) brain samples. Membranes (20 μg) were incubated as previously described without (open symbols) or with (filled symbols) 7.5 nM tau in 1 mL final volume with the assay medium, which also contained 0.01% saponin. Activities were assayed after triggering the reaction with 1 mM ATP followed by the addition of the indicated concentrations of MB. Data are expressed in µmol.min^−1^.mg^−1^ protein ±SE of four experiments performed with four preparations. * *p* < 0.001 vs. control.

In previous studies, we have shown that tau inhibits the activity of PMCA protein in human brain membranes from control and AD subjects and from other species [31]. In addition, we proved that MB was able to promote maximal enzymatic activation of PMCA and that it could block and/or prevent the inhibitory effect of the AD-associated Aβ peptide on PMCA [35]. The present study expands on our previous work by investigating if MB could also exert a similar protective effect against PMCA inhibition by tau. Thus, the effect of increasing concentrations of MB on PMCA activity was assayed in membranes from AD and age-matched control brain samples, untreated and treated with 7.5 nM tau (Figure 1). As expected, in the absence of tau, MB increased Ca^2+^-ATPase activity up to approximately 40% in both control and AD-affected membranes with similar profiles. The presence of 7.5 nM tau produced 38% inhibition of the Ca^2+^-ATPase activity, but the addition of MB (up to 100 µM) significantly enhanced the ATPase activity in both cases, reaching similar *V*max values than those obtained in the absence of tau.

### 2.2. Methylene Blue Stimulates the Purified PMCA Activity Independently of the Phospholipid Charge and Tau

**Figure 2:**
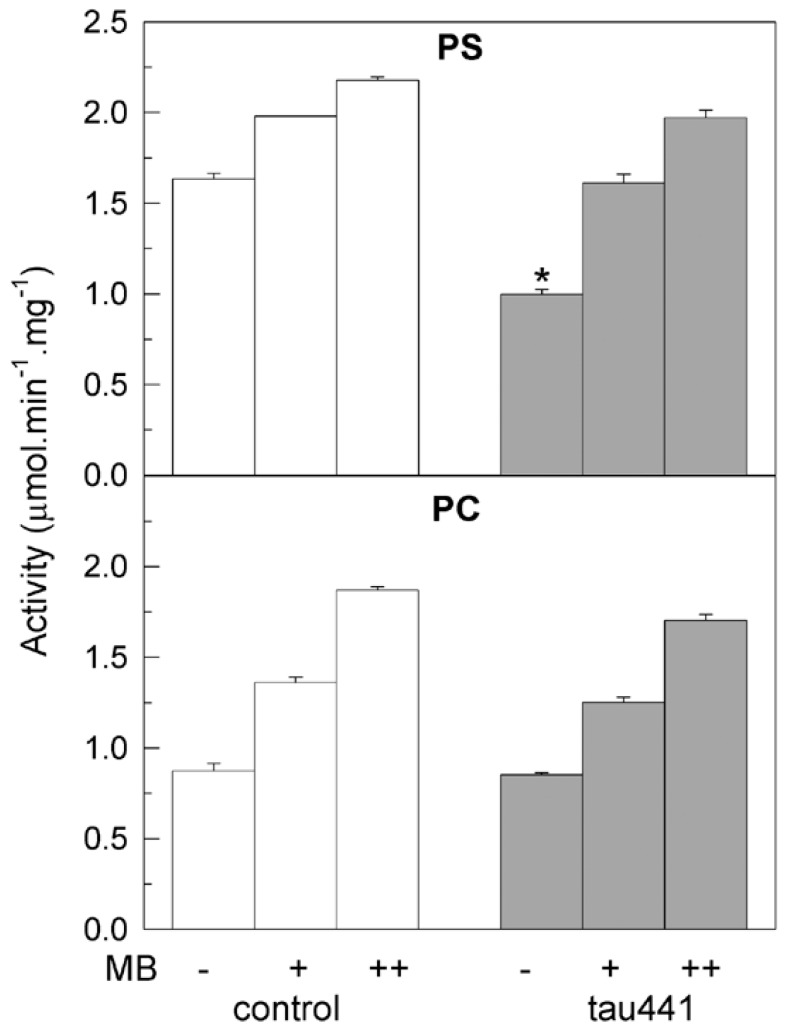
MB activates the purified synaptosomal plasma membrane Ca^2+^-ATPase (PMCA) activity and reverses the inhibitory effect of tau, independently of the lipid used for reconstitution. Purified PMCA from pig brain (2.5 μg) was mixed with 13.3 μg of phosphatidylserine (PS) or phosphatidylcholine (PC), and incubated for 2 min at 37 °C in the absence (white bars) or presence (grey bars) of 7.5 nM of tau in a 25 µL total volume, followed by the addition of up to 1 mL assay medium. The Ca^2+^-ATPase activity was measured as described in the Methods section, triggering the reaction with 1 mM ATP in the absence (−) and after subsequent additions of 50 µM MB (+) and 100 µM MB (++). Activity values are expressed in µmol.min^−1^.mg^−1^ protein mean ±SE of eight experiments performed with four preparations. * *p* < 0.001 vs. control.

We have previously established that tau inhibits the purified PMCA when is reconstituted with acidic phospholipids, but not with neutral lipids [34]. In order to know if the activating effect of MB on PMCA could be also phospholipid-dependent we did kinetic assays with purified delipidated pig brain PMCA, using acidic phosphatidylserine (PS) or neutral phosphatidylcholine (PC) to reconstitute the functional and active protein. As shown in Figure 2, the PMCA activity was higher in the presence of PS (1.6 µM/min/mg) than in PC (0.9 µM/min/mg), as previously described [32,38] and the MB dye activated the protein in both cases, reaching values of 2.3 µM/min/mg and 1.9 µM/min/mg, respectively. Similar *V*max values were reached when MB was added to the protein pre-treated with 7.5 nM tau. Therefore, the MB was able to activate PMCA independently of the lipid charge.

### 2.3. Methylene Blue Reverses the Inhibitory Effect of Tau on PMCA Isoforms Expressed in COS Cells

**Figure 3:**
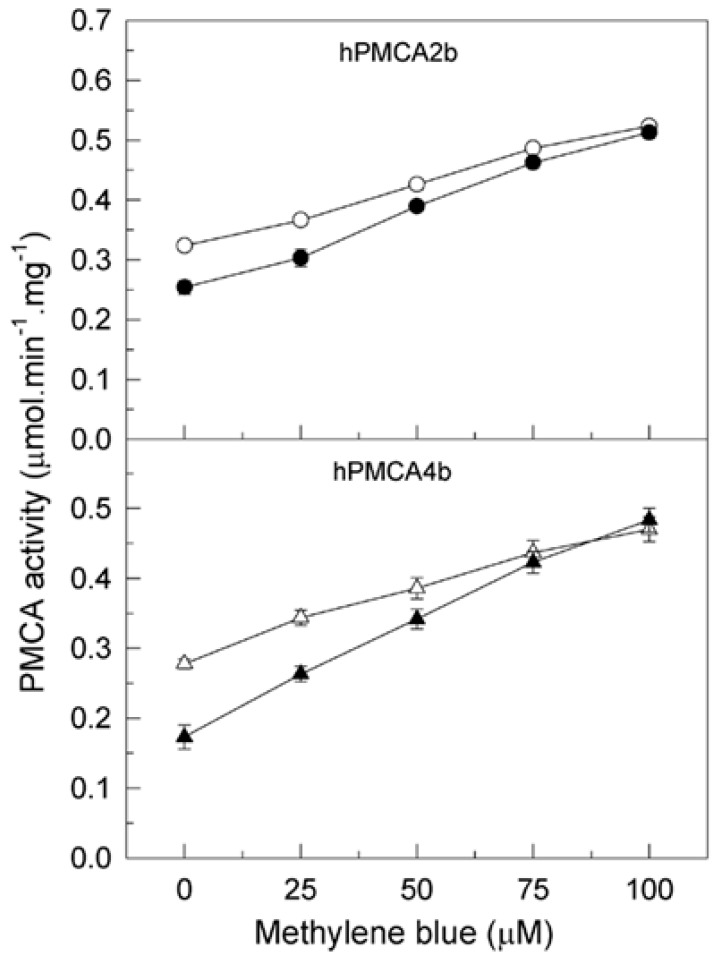
Reversion of PMCA inhibition by tau is also observed in specific isoforms. A total of 20 µg of membranes from COS cells overexpressing hPMCA2b and hPMCA4b isoforms were incubated in 1 ml final volume of assay medium, which also contained 0.01% saponin. Activity was measured after addition of 1 mM ATP and without (open symbols) or with (filled symbols) 7.5 nM tau. Successive additions of MB were added as indicated. Results are expressed in µmol.min^−1^.mg^−1^ protein as mean ±SE of four experiments performed with four preparations.

The experiments mentioned above have been done in human brain membranes, which contain the four main PMCA isoforms, as previously reported in Berrocal et al. [35], with a higher contribution of isoforms 2 and 4. In that study we also showed that MB activates both isoforms overexpressed in COS cells, independently of the sensitivity of human (h) PMCA4b but not of hPMCA2b to inhibition by Aβ. We have used the hPMCA2b splice form because it holds the full length autoinhibitory calmodulin binding domain and the C-terminal PDZ domain interacting sequence, which has been shown to play a key signaling role in synapse [39] and has a wider brain distribution than the shortest PMCA2a [40]. The hPMCA4b splice variant is used because it is highly expressed in excitable tissues [41]. Considering those observations in this work, we want to investigate if the inhibitory effect of tau is also isoform-dependent and if the presence of tau modulates the PMCA activation by MB in these isoforms. Therefore, we measured the effect of increasing concentrations of MB in the Ca^2+^-ATPase activity of COS cell membranes expressing hPMCA 2b and 4b isoforms, before and after incubation of the membranes with tau (Figure 3). As shown, in the absence of tau the MB activated Ca^2+^-ATPase activity of hPMCA2b (61.5 ± 5%) and hPMCA4b (88 ± 6%) in a dose-dependent manner, as previously reported [35]. However, 7.5 nM tau inhibited both isoforms, as inhibition is higher for hPMCA4b (41 ± 8%) than for hPMCA2b (22 ± 5%). Subsequent MB additions reversed ATPase inhibition by tau and further activated both isoforms up to the same *V*max values as the control samples.

**Figure 4:**
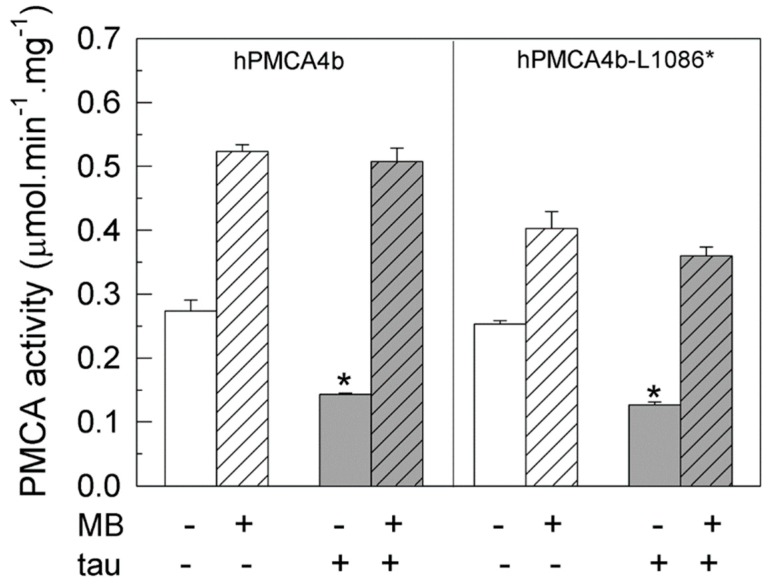
Activities of intact and truncated hPMCA4b isoforms are differentially regulated by tau and MB. Membranes (20 μg) prepared from COS-7 cells over-expressing the native human PMCA4b and truncated hPMCA4b-L1086* and hPMCA4b-R1052* isoforms were incubated, as previously described, without (white bars) or with 7.5 nM tau (grey bars). Activities were assayed by successive additions of 1 mM ATP and 100 µM MB (striped bars) as indicated and are expressed in µmol.min^−1^.mg^−1^ protein mean ±SE of four experiments performed with four preparations. * *p* < 0.001 vs. control.

Although both PMCA isoforms 2 and 4 are sensitive to tau and MB, the maximum inhibition produced by tau in PMCA4b is nearly twice the inhibition produced in PMCA2b. Thus, we analyzed in depth the effects of MB on PMCA4b inhibited by tau, focusing on the full-length protein and truncated hPMCA4b variant, and missing the calmodulin binding domain (hPMCA4b-L1086*). Figure 4 shows that both the native and truncated proteins are inhibited (~50 ± 8%) by 7.5 nM tau, corroborating previous results [34]. Nevertheless, 100 µM MB could activate the native hPMCA4b pump and the hPMCA-L1086* mutant, up to the activity values achieved in the absence of tau.

### 2.4. Methylene Blue Suppresses SH-SY5Y Cells Toxicity Caused by Exogenous Tau and Preserves Endogenous Ca^2+^-ATPase Activity

**Figure 5:**
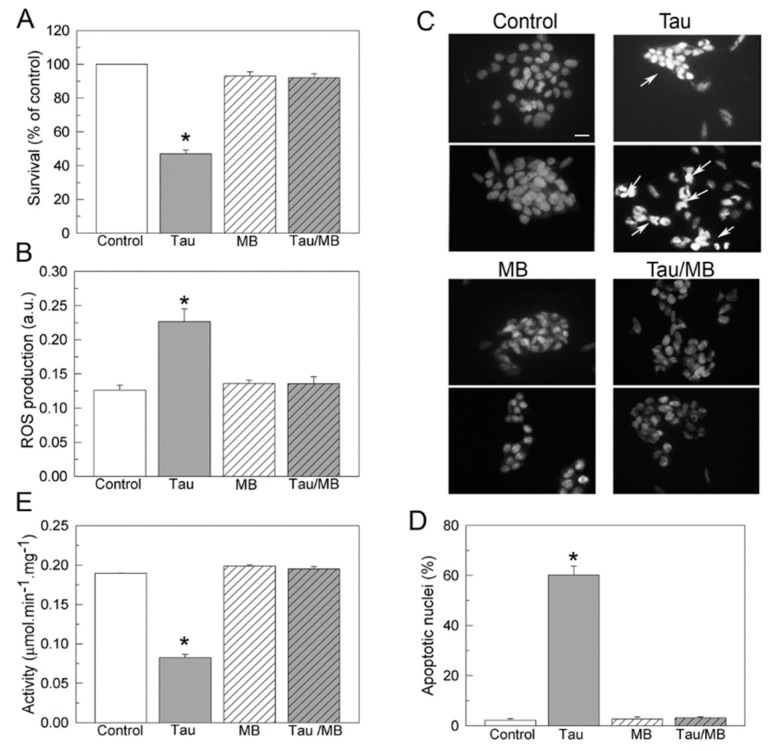
Effects of tau and MB on cell viability (**A**), ROS production (**B**), apoptosis (**C**,**D**), and Ca^2+^-ATPase activity (**E**), in human SH-SY5Y neuroblastoma cells. Cells were growing in the absence or presence of 10 nM tau, without or with 10 nM MB, as detailed in Materials and Methods. (**A**) Cell viability was measured in control and treated cells after 1 h incubation with 150 μg/mL MTT. The viability of cells treated with tau decreased to 50% of that of the control group, but increased to the same control level when co-treated with tau and MB. Values are expressed as the percentage of the untreated control cells and represented as mean ± SE of at least five independent experiments. (**B**) ROS production was assayed in control and treated cells after 30 min incubation with 40 μM H_2_DCFDA (ROS sensitive fluorescent dye). (**C**) Ca^2+^-ATPase activity was assayed in 10 μg of membranes prepared from SH-SY5Y cells, previously treated as indicated above. The total Ca^2+^-ATPase activity was measured as indicated in the method. Data represent mean ± SE of four experiments performed in duplicate from three cell cultures. (**C**) Fluorescence microscopy of DAPI staining in controls and cells incubated with tau or/and MB. The panel shows representative images of apoptotic nuclei identified by condensed chromatin and nuclear fragmentation (white arrows). Scale bar: 10 μM (**D**) Quantification of apoptotic nuclei with respect to the total number of seeded cells in each type of culture. Values are mean ± SE of ten images per coverslip, obtained from four independent cultures. * *p* < 0.001 vs control cells.

MB can passively diffuse across the cell membrane [42]. On the other hand, it has been shown that tau can also be internalized in neuronal cultures [43,44,45,46], promoting cytotoxic effects [34]. Therefore, we analyzed if MB could protect the cells from tau cytotoxicity and how it may affect the functionality of the endogenous Ca^2+^-ATPase in those cells. As shown in Figure 5A,B, the presence of 10 nM tau reduced the cellular viability by 55% and increased mitochondrial ROS production by ~60%, corroborating previous observations [34]. However, those effects were totally reversed when cells were co-treated for 24 h with 10 nM MB and 10 nM tau (Figure 5A,B). Tau cytotoxicity leads to apoptosis. The apoptotic state of cells after treatments was established by the presence of condensed nuclei stained with DAPI (Figure 5C,D). Tau exposure increases the number of apoptotic nuclei by 70%, with respect to non-treated cells. By contrast, incubation with MB or co-treatment with tau and MB reduce the percentage of apoptotic cells to the same level found in the control cells.

Ca^2+^-ATPase activity assays were performed in untreated membranes or membranes treated with 10 nM MB or/and 10 nM tau. As shown in Figure 5E, the activity of the PMCA in cells treated with tau was reduced by 50%, with respect to that found in non-treated cells or in cells treated with MB, or co-treated with 1:1 molar ratio tau and MB. Similar results were obtained by the addition of tau or/and MB in non-treated cells (not shown), indicating that MB effects are due to direct modulation of the PMCA.

### 2.5. The Interaction of Methylene Blue and PMCA Is Modulated by Tau

**Figure 6:**
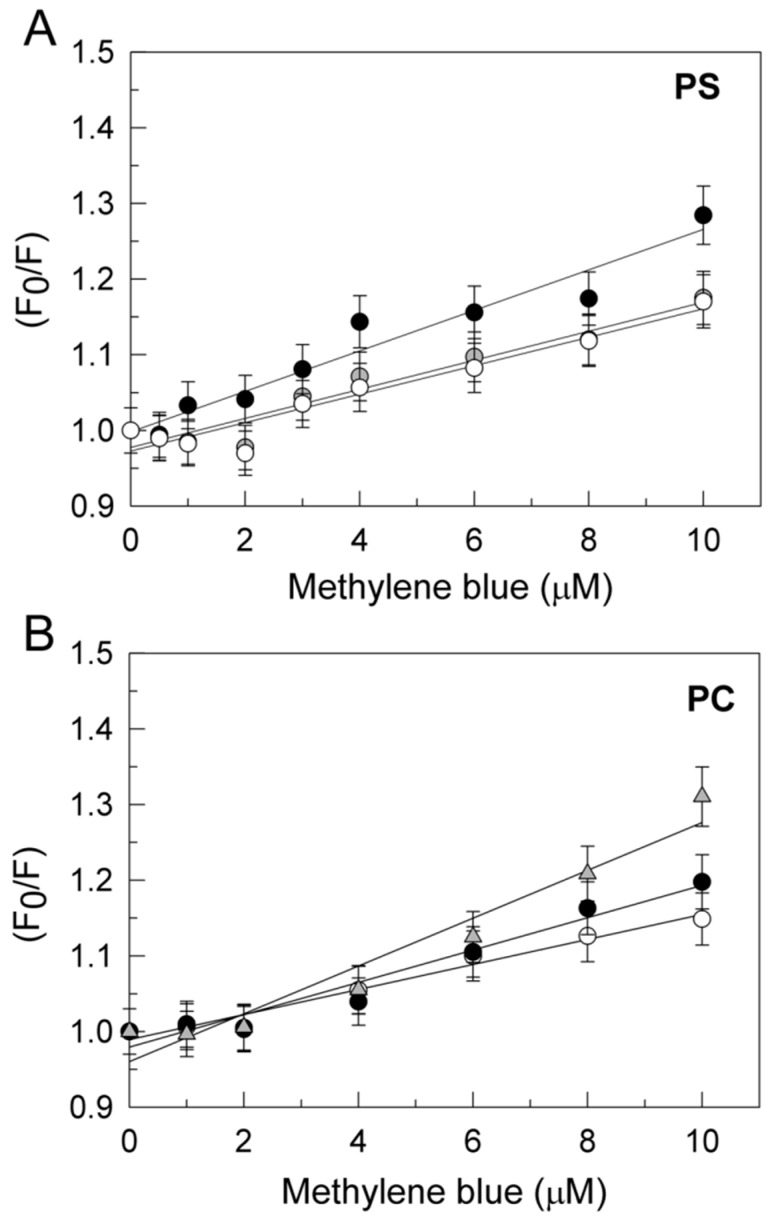
Tau modulates MB:PMCA interaction in purified pig brain PMCA. Stern–Volmer plots of the quenching by MB of the intrinsic fluorescence of 10 µg of purified PMCA from pig brain reconstituted in PS (**A**) or PC (**B**). Panel A: Results of titration with MB of PMCA reconstituted in PS in the absence of tau (**⭘**) and after incubation for 2 min at 25 °C with 0.1 nM (⬤) or 7.5 nM tau (**⬤**). Panel B: Results of titration with MB of PMCA reconstituted in PC in the absence of tau (**⭘**) and after incubation for 2 min at 25 °C with 7.5 nM (⬤) or 100 nM tau (▲). Assays were performed as indicated in the Materials and Methods in the medium used for PMCA activity measurements, but in the absence of pyruvate kinase, lactate dehydrogenase, phosphoenolpyruvate, and NADH. The inner filter effect due to the MB absorbance at 290 and 340 nm has been corrected as indicated in the Materials and Methods. Data shown are means ± SE of titration data of three experiments performed with three preparations.

Proteins display intrinsic fluorescence due to aromatic residues (mainly tryptophan) that are very sensitive to their local environment. In fact, the fluorescence of a protein usually changes as a result of its interaction with other molecules, in a concentration dependent manner. Therefore, this method can be used to analyze protein–ligands interactions. In a previous work we found that MB binding to PMCA can be monitored by quenching of the PMCA intrinsic fluorescence in the presence and absence of Aβ and that the dissociation constant of MB with PMCA is modulated by the conformational state of PMCA [35]. In order to investigate whether MB–PMCA interaction could also be modulated by tau we performed a PMCA fluorescence titration with MB in the presence of a fixed tau concentration in the assay medium used for activity measurements. It is to be noted that tau intrinsic fluorescence intensity (up to 100 nM tau) is negligible relative to the intrinsic fluorescence of 10 µg of purified PMCA from pig brain, i.e., <5% of PMCA intrinsic fluorescence. Considering that the effect of tau on PMCA activity is strongly dependent on the ionic nature of the phospholipid and was only seen with negatively charged lipids [34], the fluorometric study was done with PMCA previously reconstituted in PC or PS separately. Stern–Volmer plots of the MB-induced quenching of the intrinsic fluorescence of PMCA reconstituted in either PC or PS are shown in the Figure 6. On the other hand, tau (up to 100 nM) did not elicit a significant change in the fluorescence spectrum of 100 nM MB (data not shown), excluding a significant complexation of MB by tau. The Stern–Volmer constant (*K*_SV_) and the dissociation constant of MB:PMCA complexes (*K*_d_) derived from these results are given in the Table 1. Direct plots of the quenching of fluorescence versus MB concentration fit well to the one-site binding equation with these *K*_d_ values (Supplementary Figure S1), yielding between 60% and 80% quenching of fluorescence at MB saturation. However, the high inner filter effect above 10 µM MB, due to the absorbance of MB, did not allow us to perform more precise measurements of the quenching of fluorescence at MB saturation. It is to be noted that the dissociation constants obtained from these Stern–Volmer plots are close to the MB concentration needed to produce a 50% reversion of PMCA inhibition by tau (Figure 1). The presence of 7.5 nM tau, a concentration that inhibits by 50% the PMCA activity in PS but did not have any effect on PMCA in PC, has an effect on the fluorescence quenching produced by MB in PMCA reconstituted in PS stronger than in PMCA reconstituted in PC. It was necessary to increase tau up to 100 nM to produce a quenching of PMCA fluorescence in PC by MB, close to the one obtained by 7.5 nM tau with PMCA in PS. These results indicate that the affinity of PMCA for MB is very similar when PMCA is reconstituted in PS or in PC and that tau potentiates the quenching of intrinsic PMCA fluorescence-elicited MB binding to PMCA, with nearly 10-fold greater potency when PMCA is reconstituted using PS instead of PC.

Taking into account that the purified pig brain PMCA is a mixture of isoforms [35], the fluorometric titration with MB was also done with the purified hPMCA4b expressed in yeast [47]. The results obtained upon titration with MB of the intrinsic fluorescence of yeast hPMCA4b reconstituted in PS and PC were very similar to those obtained with purified PMCA from pig brain, both in the absence or in the presence of tau (Table 1 and Supplementary Figure S1). Indeed, *K*_d_ values of MB:hPMCA4b and MB:PMCA from pig brain, reconstituted in PC and in the absence of tau, were nearly identical within experimental errors. The most significant differences are observed between the *K*_d_ values of MB:hPMCA4b and MB:PMCA from pig brain reconstituted in PS and the higher sensitivity of the *K*_d_ (MB:hPMCA4b) to low concentrations of tau with respect to PMCA from pig brain reconstituted in PS.

## 3. Discussion

In this paper we report on our progress so far on the beneficial action of MB on the functional alteration of PMCA protein by molecular markers of AD. In a previous work we showed a similar effect of MB upon the toxic effect of Aβ1-42 on PMCA activity. Here, we provide functional evidence that MB modulates the toxic effect of tau on PMCA in brain tissues from human control and AD subjects, in pig brain, and in cell cultures. Our kinetic studies clearly reveal both full restorations of PMCA activity by MB after inhibition by tau and protection by MB against the inhibitory effect of tau on PMCA. Thus, MB can fully revert the inhibition of PMCA by the AD hallmarks Aβ and tau. As a consequence of the major role of PMCA in maintaining cytosolic calcium homeostasis in neurons [48,49,50,51], these results indicate that MB can act as a drug that affords protection against the neurotoxicity of the sustained rise of cytosolic calcium induced by Aβ and tau, thereby slowing down brain neurodegeneration induced by these major AD biomarkers. The MB effects were seen at very low concentrations of tau (around 7.5 nM). These findings identified novel mechanisms of action by MB as an AD drug.

Tau-mediated neurodegeneration can arise from the loss of physiological function and/or the gain of toxicity, but the mechanism underlying neurodegeneration induced by tau is unclear. It has been extensively proposed that tau aggregation is a key factor for tau-induced toxicity [52], although growing evidence suggests that neuronal toxicity might be induced by soluble tau species, including oligomers [53,54]. Furthermore, it has been reported that the mechanism of protein aggregate-induced toxicity is cell membrane disruption, resulting in the alteration of ion homeostasis and dysregulation of neuronal signal transduction [55,56,57,58].

A strong interaction of human tau with anionic lipid membranes, which stimulate protein–protein interactions leading to protein aggregation and membrane disruption, been observed, using X-ray and neutron scattering techniques [57] or fluorescence correlation spectroscopy [59].

In our previous work, we showed that tau inhibits the PMCA activity only in the presence of acidic phospholipids such as PS [35]. In this work we show that MB is able to completely revert this inhibition. Since 1996 it is known that MB is able to inhibit tau aggregation in vitro by blocking protein–protein interaction [20]. Since then, this dye has attracted increasing interest because of its use as a therapy to improve cognitive functions in AD patients [11,60,61], based on the fact that the formation of tau aggregates is involved in its toxicity and that inhibition of tau aggregation by MB may be linked to a reduction or loss of toxicity [62]. As aforementioned, it is under debate if soluble, oligomeric, or insoluble aggregated tau forms represent the toxic species. In fact, it has been reported that monomeric tau is able to induce the spread of tau pathology [63] and that soluble tau (composed mostly of monomers and dimers) produces toxic effects on the plasticity and connectivity of hippocampal granule neurons [64]. However, as discussed elsewhere [62], MB may use different mechanisms of action, besides inhibition of tau aggregation, to produce its therapeutic effects. In fact, it has been shown that MB may improve cognitive functions in transgenic mice by increasing proteasome function [65]. Additionally, MB can induce autophagy to reduce tau levels in cell models and brain slices [66]. In line with these findings, our results suggest that MB must also use another protective mechanism focused on preventing PMCA protein from being inhibited by tau, as the subsequent addition of MB to the assay medium containing the protein inhibited by tau resulted in the instant full recovery of PMCA activity. Analogous MB effects were previously observed on the PMCA inhibited by Aβ1-42, the other significant molecule involved in the pathology of AD [35].

Besides accumulation of abnormally aggregated Aβ and tau, a significant feature of AD pathology is oxidative damage and mitochondrial dysfunction [67]. On the other hand, although tau is an intracellular axonal protein, it has been proposed that extracellular tau can also cause toxic effects [63,68,69]. In cultured SH-SY5Y cells, exogenous tau promotes the phosphorylation of intracellular tau and its release to the extracellular medium, resulting in cell death. This effect is mediated by the activation of muscarinic receptors and a tissue-nonspecific alkaline phosphatase. After cell death, the soluble tau may be released from the neuron and stimulate intracellular calcium increase [68,70,71,72]. These authors have proposed this mechanism for tau propagation and toxicity in AD pathology. We have recently reported that nanomolar concentrations of tau reduced cell viability, increased mitochondrial ROS levels, and inhibited PMCA activity, and that calmodulin, an endogenous PMCA activator, protected both the cells and the endogenous PMCA activity from tau toxicity [34]. In the present study we observed that the MB phenothiazine, at the concentration used in our work, exerts a full level of protection against the cytotoxic effect of tau and also protects PMCA from tau inhibition. Similar effects were seen when tau and MB were added to the assay medium containing membranes from untreated cells.

In a previous work, we have shown that the effect of MB in the intrinsic fluorescence of PMCA can be used to monitor the binding of MB to PMCA [35]. The emission peak of the intrinsic fluorescence of PMCA is between 335 and 340 nm, pointing out that it is largely due to Trp fluorescence emission.

Of note, it has been shown with other proteins that MB affords a large quenching of Trp fluorescence upon the binding of MB to protein sites close to accessible Trp(s), mainly by static quenching [73,74]. In the presence of tau, the MB-induced quenching of the intrinsic fluorescence of PMCA purified from pig brain or purified hPMCA4b reconstituted in PS or PC is largely enhanced with a significant increase of PMCA affinity for MB. As tau (up to 100 nM) does not alter the fluorescence of MB, significant complexation of MB with free tau is unlikely in our experimental conditions. On these grounds, the increase of the quenching of the PMCA intrinsic fluorescence by MB in the presence of tau can be rationalized in terms of long-range conformational changes, because the tryptophans of this protein are widely scattered in the primary amino acids sequence (7 of them located in transmembrane α-helix domains, 1 in the extracellular domain connecting TM3 and TM4, and 4 in cytosolic domains) (source: UniProtKB: locus AT2B4_BOVIN, accession D3K0R6).

Of note, the concentration of tau that produces a significant increase of the MB-induced quenching of the intrinsic fluorescence of PMCA is strongly dependent on the lipid used for PMCA reconstitution, being nearly 10-fold lower when purified pig brain PMCA or purified yeast hPMCA4b are reconstituted in PS instead of in PC. This is fully consistent with the dependence of the inhibitory effect of tau on PMCA activity on the phospholipid used for reconstitution. As shown in a previous work [34], tau inhibits the ATPase activity only when purified PMCA is reconstituted in PS or in binary mixtures of PS:PC containing more than 25% PS, but not when is reconstituted in PC. It is worth recalling that the ATPase activity of PMCA in the brain is strongly stimulated by PS [38], that tau binds to a site of the C-terminal domain of PMCA close to the acidic lipids binding site [34] and also with high affinity to acidic phospholipids, but not to neutral phospholipids [57,75,76]. An increase of PS content in brain membranes of AD with respect to control age-matched subjects has been reported [77,78].

Finally, the dissociation constants obtained in this work for the PMCA:MB complex from titration of the intrinsic fluorescence with MB, either for purified PMCA from pig brain or for hPMCA4b reconstituted in PS or in PC in the presence and absence of tau, are close to the concentrations of MB that afford a 50% reversion of inhibition of PMCA activity by tau. It is to be noted that tau increases the affinity of MB for PMCA, such that only low micromolar MB concentrations need to be reached in the brain to have significant binding of MB to brain PMCA in vivo. Indeed this seems to be the case, because it has been shown that MB raised up to 5–10 µM in human blood plasma after a therapeutic intravenous bolus injection of 1.4 mg MB/kg body weight [36] and pharmacokinetic studies in rodents have shown that MB permeates across the blood-brain barrier and displays a rapid and extensive accumulation in the nervous system [5,37].

In conclusion, MB binds to PMCA at micromolar concentrations and can reverse the inhibition produced by tau in the ATPase activity of PMCA of human brain membranes prepared from AD and age-matched control samples, purified pig brain PMCA, or purified yeast hPMCA4b reconstituted in PS or PC, and membranes of COS cells overexpressing hPMCA2b or hPMCA4b. Furthermore, a concentration as low as 10 nM methylene blue prevents the toxic effects of tau in neuroblastoma SH-SY5Y cells and the inhibition of the endogenous Ca^2+^-ATPase activity. As these effects are observed at concentrations of MB that are reached in the nervous system after therapeutic injections, brain PMCA modulation by MB is likely contributing to the beneficial effects of this drug in AD.

## 4. Materials and Methods

Phosphatidylcholine (PC) type XI-E from egg yolk, phosphatidylserine (PS), calmodulin-agarose, and methylene blue (MB) were obtained from Sigma. Methylene blue was prepared as stock solution of 5 mg/mL (w/v) and made freshly for each separate experiment. Tau 441 was obtained from Enzo Life Sciences. Pig brains were obtained from a local slaughterhouse and human autopsy brain tissues (medium frontal gyrus, a brain region highly affected in AD) were supplied by the Netherlands Brain Bank (NBB, Netherlands Institute for Neuroscience, Amsterdam, Netherlands) and obtained from 8 neuropathologically confirmed AD patients (age 79 ± 2yr, Braak stage V/VI) and from 7 age-matched non-diseased control subjects. Procedures, information, and consent forms of the NBB have been approved by the Medical Ethics Committee of the Vrije Universiteit Amsterdam Medical Centre on 30 April 2009. The project identification code is Avila(537). Investigations using human tissues were carried out following the rules of the Declaration of Helsinki of 1975 (https://www.wma.net/what-we-do/medical-ethics/declaration-of-helsinki/), revised in 2013.

### 4.1. Preparation of Membrane Extracts from Cells and Human Brain Tissues

Native human PMCA4b (hPMCA4b), truncated hPMCA4b-L1086* (which lacks the calmodulin-binding domain), and hPMCA4b-R1052* (which lacks the calmodulin-binding domain and the putative ethanol binding site) [79], were expressed in fibroblast-like monkey COS-7 cells (ATCC, Rockville, MD, USA), as described in Berrocal et al. [33,34]. Membrane extracts were prepared following the protocol described by Sepulveda et al. [80]. Briefly, tissues were excised and cells were scraped and pelleted. Tissues and cell pellets were homogenized in 10 mM HEPES/KOH, pH 7.4; 0.32 M sucrose; 0.5 mM MgSO_4_; 0.1 mM phenylmethanesulfonyl fluoride (PMSF); 2 mM 2-mercaptoethanol; and protease inhibitor cocktail solution (Roche Diagnostics, Mannheim, Germany). Homogenates were centrifuged for 10 min at 1500 g and supernatants were centrifuged for 45 min at 100,000 g. The final pellets were resuspended in 10 mM HEPES/KOH, pH 7.4 and 0.32 M sucrose, aliquoted and stored at −80 °C until use. The protein content was evaluated by the Bradford method [81].

### 4.2. Preparation of Purified Plasma Membrane Ca^2+^-ATPase from Pig Brain and Yeast

The pig brain plasma membrane Ca^2+^-ATPase (PMCA) was purified from synaptosomal plasma membrane vesicles as described in detail by Salvador and Mata [38]. Native hPMCA4b and truncated hPMCA4b (hPMCA4b*) versions (similar to COS hPMCA1086*) were expressed and purified from *S. cerevisiae* as detailed in Corbacho et al. [82]. Samples were stored at −80 °C until use.

### 4.3. Ca^2+^-ATPase Activity Assays

The activity was measured with a coupled enzyme assay, as described in Salvador and Mata [38], using 20 µg membrane vesicles from brain tissue from Alzheimer’s disease patients and control subjects and from COS-7 cells or 2.5 µg of delipidated purified PMCA from pig brain reconstituted with 5.33 µg of PS or PC. The effect of tau on purified PMCA was assayed by incubating for 2 min at 37 °C with 300 nM tau441, in a final volume of 25 µL. The mixture was then diluted directly into the assay medium (50 mM Hepes/KOH (pH 7.4), 100 mM KCl, 2 mM MgCl_2_, 5 mM NaN_3_, 3.16 µM free Ca^2+^ (pCa 5.5), 0.22 mM NADH, 0.42 mM phosphoenolpyruvate, 10 IU pyruvate kinase, and 28 IU lactate dehydrogenase) in 1 mL final volume, using a standard 1 cm path-length cuvette. The medium assay used for membrane vesicles also included 0.01% saponin (to disrupt both the plasma and intracellular membranes, allowing the access of substrates to all protein molecules in membrane vesicles). In the case of membrane vesicles, tau was added to cuvette at the indicated concentration. The total Ca^2+^-ATPase activities were determined in membrane preparations as described in Sepulveda et al. [83]. Briefly, the reaction was started with 1 mM ATP, followed by the addition of 7.5 nM tau or MB at the indicated concentrations, and 3 mM EGTA (to measure Mg^2+^-ATPase activity). The activity of purified pig brain PMCA was directly measured after triggering the reaction with 1 mM ATP and, when indicated, after the addition of MB at the indicated concentrations. The putative contribution of NADH oxidation by MB in our ATPase assays was experimentally assessed as follows. We recorded the absorbance change at 340 nm in the ATPase assay medium in the absence and presence of MB concentrations ranging from 10 to 150 µM, in the absence of the added biological sample. The absorbance was recorded 5 min before ATP addition and also 5 min after ATP addition, because the ATPase assay of our samples is started by addition of ATP and the change of absorbance is measured over less than 5 min, with absorbance changes per min ranging between 0.070 and 0.100 OD/min for the different samples studied herein (brain homogenates, membranes, or purified PMCA). The slow kinetics of NADH oxidation produced by MB contributed less than 10% of the total NADH oxidation rate measured with the different samples studied, up to 100 µM MB and between 11 and 15% with 150 µM MB. This contribution was already subtracted in the results shown in this manuscript. Furthermore, parallel control kinetic measurements at 665 nm, the absorption peak of MB, showed that, during the time period used for ATPase measurements in this work, leuco-MB did not accumulate and the change of MB concentration was negligible.

### 4.4. Fluorescence Measurements

Fluorescence measurements were performed using a Cary Eclipse fluorescence spectrophotometer (Agilent Technologies, Santa Clara, CA, USA) at 25 °C in 1 cm quartz cells with both excitation and emission slits of 10 nm. MB titrations were performed with 10 µg of purified pig brain PMCA or purified hPMCA4b expressed in *S. cerevisiae* previously incubated with tau over 2 min, when indicated. The assay was performed in a buffer containing 50 mM HEPES-KOH (pH 7.4), 100 mM KCl, 2 mM MgCl_2_, 3.16 µM free calcium, and 1 mM ATP. The fluorescence intensity was measured with excitation and emission wavelengths of 290 nm and 340 nm, respectively. Inner filter effects were corrected as indicated in Lakowicz [84]. Briefly, the absorbance at excitation and emission wavelengths (OD_Exc_ and OD_Em_, respectively) of the solutions used in fluorescence measurements were measured and then the corrected fluorescence intensity (F_corr_) was calculated from the observed fluorescence intensity (F_obs_) using the following equation:

F_corr_ = C · F_obs_, where C = antilog [(OD_Exc_ + OD_Em_)/2].

Quenching of the intrinsic PMCA fluorescence by MB was analyzed using the Stern–Volmer equation [84]: F_0_/F = 1 + *K*_SV_ ·[Q], where F_0_ and F are the fluorescence intensities in the absence and presence of quencher (Q = MB) concentration, *K*_SV_ is the Stern–Volmer constant, which in this case is the association constant for the complex MB:PMCA and, therefore, *K*_d_ (MB:PMCA) = 1/*K*_SV_. The MB emission spectrum (peak emission wavelength = 683 nm) was recorded with an excitation wavelength of 600 nm. No significant increase of light scattering at 420 nm was detected up to 10 µM MB, the maximum concentration of MB used in fluorescence measurements, excluding the formation of MB micelles and the need for other corrections of fluorescence intensity.

### 4.5. Neuroblastoma Cell Cultures and Treatments

The *SH-SY5Y* human neuroblastoma cell line was provided by Sigma-Aldrich. Cells were grown in Dulbecco’s modified Eagle´s medium (DMEM) supplemented with 2 mM L-glutamine, 100 mU/mL penicillin, 0.1 mg/mL streptomycin, and 10% heat-inactivated fetal bovine serum (FBS). Cells were maintained at 37 °C and incubated in humidified atmospheric air containing 5% CO_2_. After 1 day, the cells were exposed to 10 nM tau and/or 10 nM MB for 24 h. Afterward, The Ca^2+^-ATPase activity was also assayed in membranes prepared from SH-SY5Y cells, as described above.

### 4.6. Cell Viability Assay

Human neuroblastoma SH-SY5Y cells were plated overnight at a density of 10,000 cells/well in 96-well cell culture plates. Afterward, the cells were exposed to 10 nM tau and/or 10 nM MB for 24 h. Cell viability was determined by a colorimetric mitochondrial dehydrogenase activity assay according to [85]. Briefly, cells were incubated with 150 µg/mL 3-(4,5-dimethylthiazol-2-yl)-2,5-diphenyltetrazolium bromide (MTT) in phosphate-buffered-saline (PBS) for 1 h at 37 °C. The media was removed and 100 µL DMSO was added to each well. Metabolically active cells reduced MTT to a formazan precipitate that was solubilized with DMSO and quantified at 490 nm, with background subtraction at 650 nm, in a Varioskan Flash fluorescence spectrophotometer (Thermo Scientific, Madrid, Spain).

### 4.7. Reactive Oxygen Species Assay

Intracellular Reactive Oxygen Species (ROS) production was detected by using the fluorogenic dye 2′7′-dichlorodihydrofluorescein diacetate (H_2_DCFDA; Molecular Probes). After diffusion into cells, it is deacetylated by cellular esterases to a non-fluorescent compound, which is later oxidized by ROS into the fluorescent 2′7′-dichlorodihydrofluorescein (H_2_DCF). SH-SY5Y cells were seeded overnight at a density of 10,000 cells/well in 96-well cell cultured plates and next treated with 10 nM tau and/or 10 nM MB for 24 h. After removing, the medium cells were added to 40 µM H_2_DCFDA in PBS and incubated for 30 min at 37 °C under air supplemented with 5% CO_2_. Thereupon, cells were washed with PBS buffer and 25 µL NaOH 1M was added to extract the fluorescence product [86]. Cells were collected with 175 µL PBS buffer and fluorescent intensity of cell extracts (due to ROS production) was measured in a Varioskan Flash fluorescence spectrophotometer (with excitation and emission wavelengths of 485 nm and 530 nm).

### 4.8. Quantification of Apoptotic Cells

Cells, previously treated without and with 10 nM tau and/or 10 nM MB, were fixed and labeled with 0.3 µM DAPI for nuclear staining. Then, cells were identified morphologically as non-apoptotic cells, characterized by a homogeneous distribution of DNA in a normal size or as apoptotic cells showing condensed chromatin. [87,88].

### 4.9. Statistical Analysis

Significant differences were determined by an unpaired Student t-test. Non-linear regression fits, statistical analysis, and the plotting of fluorescence assay data was done using SigmaPlot v10 software (SPSS Inc, Chicago, IL). A value of *p* ≤ 0.01 was considered statistically significant.

## Figures and Tables

**Table 1:** Quenching by MB of the intrinsic fluorescence of purified pig brain PMCA (brainPMCA) and of purified yeast hPMCA4b, reconstituted in PS or PC. Fluorescence measurements were performed as indicated in the Materials and Methods and data were analyzed by linear regression fit to the Stern–Volmer equation (*R*^2^ > 0.93 in all cases).

Sample	[Tau] (nM)	*K*_SV_ (µM^−1^)	*K*_d_ (µM)
BrainPMCA-PS	0	0.019 ± 0.001	52.6 ± 2.5
	0.1	0.019 ± 0.001	52.6 ± 2.5
	7.5	0.027 ± 0.001	37.0 ± 1.9
BrainPMCA-PC	0	0.0165 ± 0.001	60.6 ± 3.2
	7.5	0.021 ± 0.001	47.6 ± 2.3
	100	0.032 ± 0.001	31.3 ± 1.5
hPMCA4b-PS	0	0.014 ± 0.001	71.4 ± 3.7
	0.1	0.0275 ± 0.001	36.4 ± 2.6
	7.5	0.035 ± 0.001	28.6 ± 1.3
hPMCA4b-PC	0	0.019 ± 0.001	52.6 ± 2.8
	7.5	0.019 ± 0.001	52.6 ± 2.7
	100	0.030 ± 0.001	33.3 ± 1.9

## Supplementary Materials

Supplementary materials can be found at https://www.mdpi.com/1422-0067/20/14/3521/s1. Supplementary Figure S1.: Quenching (Q) of the intrinsic fluorescence of PMCA reconstituted in phosphatidylserine (PS) or phosphatidylcholine (PC) by methylene blue (MB) in the absence and presence of tau. (A) Purified pig brain PMCA. (B) Purified hPMCA4b. PMCA reconstituted in PS: (**⭘**), without tau; (⬤), 0.1 nM tau; (**⬤**), 7.5 nM tau. PMCA reconstituted in PC: (**⭘**), without tau; (⬤), 7.5 nM tau; (**⬤**), 100 nM tau. The lines are the NLS fits to the hyperbolic one-site binding equation: Q = Qmax·[MB]/(*K*d + [MB]), with the Kd values listed in the Table 1. Qmax, the quenching at saturation of MB ranged between 60 and 80% for NLS fits of panels A and B. The results shown are the average ±SE of triplicate experiments.
