# Supplementary material for: Methylene Blue Blocks and Reverses the Inhibitory Effect of Tau on PMCA Function
Source: Int J Mol Sci. 2019 Jul 18;20(14):3521. doi: 10.3390/ijms20143521 (PMC6678728; doi:10.3390/ijms20143521)
Supplement: Supplementary file 1 [file ijms-20-03521-s001.pdf]

International Journal of
Molecular Sciences

MDPI

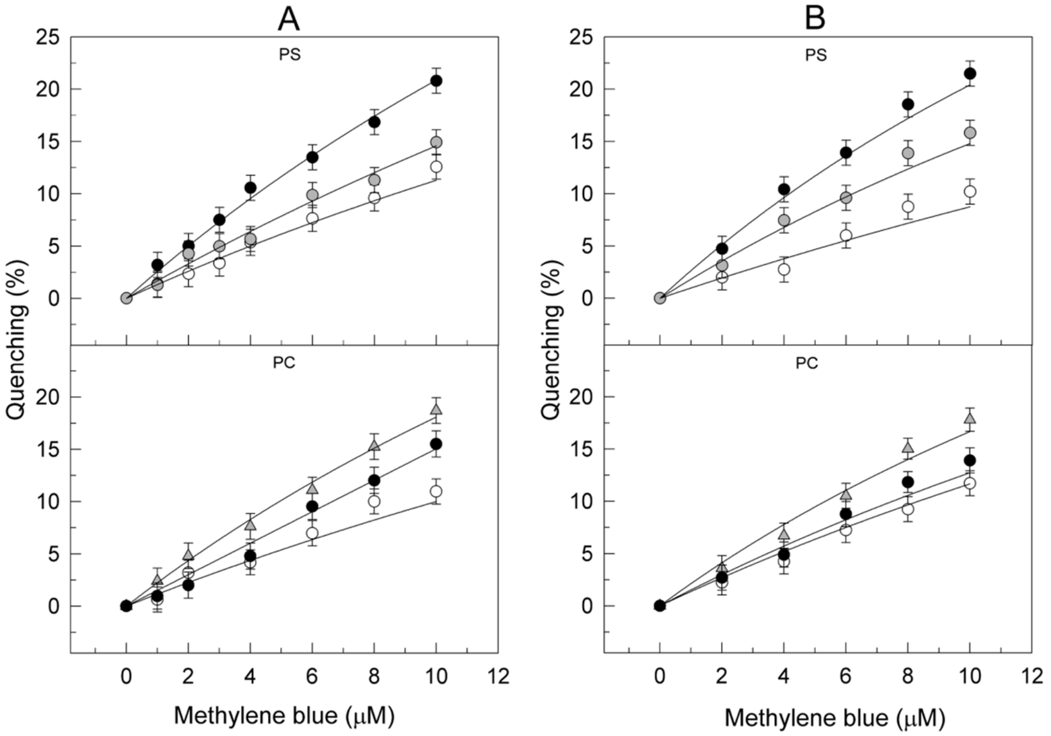

Supplementary Figure 1. Quenching (Q) of the intrinsic fluorescence of PMCA reconstituted in phosphatidylserine (PS) or phosphatidylcholine (PC) by methylene blue (MB) in the absence and presence of tau. (**A**) Purified pig brain PMCA. (**B**) Purified hPMCA4b. PMCA reconstituted in PS, without tau (○) and with 0.1 nM tau (●) or 7.5 nM tau (●). PMCA reconstituted in PC, without tau (○) and with 7.5 nM tau (●) or 100 nM Tau (▲). The lines are the NLS fits to the hyperbolic one-site binding equation: Q = Qmax·[MB]/(*K*d +[MB]), with the K_d values listed in the Table 1. Qmax, the quenching at saturation of MB ranged between 60 and 80% for NLS fits of panels A and B. The results shown are the average ±SE of triplicate experiments.
